# Nutritional and nutraceutical potential of rice bean (*Vigna umbellata*) –a legume with hidden potential

**DOI:** 10.3389/fnut.2023.1126544

**Published:** 2023-06-09

**Authors:** Rajan Katoch, Sanjay Kumar Sanadya, Kiran Pathania, H. K. Chaudhary

**Affiliations:** Department of Genetics and Plant Breeding, Chaudhary Sarwan Kumar Himachal Pradesh Krishi Vishvavidyalaya, Palampur, India

**Keywords:** rice bean, *Vigna*, nutritional quality, nutritional security, protein fractionation

## Abstract

In the recent years there has been paradigm shift in global agriculture for the exploration of different underutilized crops as future potential crops. Rice bean [*Vigna umbellata* (Thunb.) Ohwi and Ohashi] one of the lesser known pulses among *Vigna* species has gained attention during last decade as food and nutritional security crop. Rice bean seeds are well-balanced source of beneficial constituents such as protein, carbohydrates, minerals, vitamins, polyunsaturated fatty acids (PUFAs) and anti-oxidants for health benefits and combating malnourishment in human. In the present investigation, seeds of 15 diverse rice bean accessions from north-western Himalayan region were analyzed for nutrients, anti-nutrients and nutraceutical traits. Significant differences were observed among genotypes for different traits. The rice bean genotypes revealed variation for major quality traits including total carbohydrates (50.56–56.87%), crude protein content (22.56–25.97%) and lipid content (1.87 to 3.17%) with the higher proportion of linolenic acid followed by linoleic acid which are nutritionally desirable PUFAs. The genotype IC-548758 revealed higher proportion of desirable quality traits. Among protein fractions, globulins and albumins constituted major seed storage protein fraction in rice bean seeds. The wide range variation was also observed for anti-nutrients like including raffinose family oligosaccharides (RFOs), phenolics, tannins, trypsin inhibitor (TI), phytic acid, lipoxygenase activity and saponin content among genotypes. Insignificant correlation among iron, zinc, magnesium and manganese revealed good selection accuracy for genetic biofortification program in rice bean. In summary, the genotype IC-548757, IC-548760 and IC-548770 revealed lower proportion of anti-nutrients, whereas, the genotype IC-548759 and IC-548757 revealed higher level of free radical scavenging activity indicating nutritional and nutraceutical superiority of these genotypes. Overall, the study revealed nutritional superiority of genotype IC-548770, IC-548758 and IC-548760 with balanced proportions of nutrients and anti-nutrients. Rice bean legume has the potential to support more sustainable and resilient food and nutritional security in future. Our study highlights the potential of different rice bean genotypes as functional ingredients for future food and nutritional security programmes.

## Introduction

Food and nutrition security programmes are expected to face a tough challenge in coming future due to population outburst especially in the developing countries. The present agricultural system, which focuses on a bunch of primary food crops (such as rice, wheat and maize), was successful in achieving food security in the past, but in 21th century it is facing multiple challenges (1). This and several other issues call for a paradigm shift in agriculture system to explore non-conventional pathways such as adoption and promotion of neglected and/or underutilized crops species (NUS). These crops possesses agronomic, nutritional and climate resilient traits and suite to wide range of climates (2–6). They have the potential to improve food availability and generate rural economy through the development of new value chains (1, 6–8). Therefore, to mitigate the global food and nutritional insecurity, there is a need to explore these potential food resources in future. Underutilized legumes are rich in phytochemicals, antioxidants and phenols and are capable of promoting health status that could help preventing different ailments in humans (9). Rice bean is one of such underutilized crop which could be best exploited for food, nutritional and nutraceutical potential.

Rice bean [*Vigna umbellata* (Thunb.) Ohwi and Ohashi], a potential underutilized crop is of current interest in view of its production potential and nutritive profile with high protein content, amino acid composition, appreciable level of micronutrients and anti-oxidants (10). The legume was originally cultivated by farming communities for sustenance and livelihood, thereby its cultivation was earlier restricted to specific agro-ecological regions (1, 11). It is a native of South and South-East Asia and usually grown in remote areas. The crop also has favorable agronomic attributes, high nodulation efficiency and has capacity to thrive even in harsh climatic conditions (1, 12). Rice bean in vegetative stage also provide considerable amount of palatable and nutritious fodder. The pulse has recently drawn attention as potential source of quality proteins and other essential nutrients for bridging “protein gap” (13). In terms of nutritional value, rice bean is comparable to other low-fat grain pulses. The seeds are well-balanced source of nutrients such as proteins (methionine and tryptophan), carbohydrates, lipids, vitamins (niacin, riboflavin, thiamine and ascorbic acid), minerals and unsaturated fatty acids (linolenic, linoleic and oleic acids) (14–16). Theseeds have appreciable levels of flavonoids and antioxidant potential possessing free radical-scavenging activity among common pulses in *Vigna* group. Interestingly, rice bean has significant α-glucosidase inhibition (AGI) activity reflecting its potential to suppress the blood glucose by inhibiting α-glucosidase in diabetic persons. Rice bean thereby hold merits in nutritional and nutraceutical attributes (11). Rice bean has also variable levels of antinutrients that affect the absorption of nutritionally desirable constituents.

Being underexploited crop, there is still meager information available on different quality and anti-quality attributes of rice bean. Our laboratory is pioneer in undertaking research on rice bean legume from more than last two decades and has exposed its hidden attributes globally that now has attracted attention of different research groups for advance research in rice bean genomics and proteomics (17–19). The nutritional profile of several rice bean genotypes has been reviewed based on our observations for addressing the food and nutritional issues. The current investigation was focused on the nutritional and nutraceutical attributes of rice bean genotypes explored from remote locations in north-western Himalayan region.

## Materials and methods

### Experimental materials

Rice bean germplasm was collected from the diverse locations of north-western Himalayan region and was submitted with National gene bank (NBPGR, New Delhi, India) for procuring the germplasm accession number. Total of 15 rice bean genotypes *viz.*, IC-548756, IC-548757, IC-548758, IC-548759, IC-548760, IC-548761, IC-548762, IC-548763, IC-548764, IC-548765, IC-548766, IC-548767, IC-548768, IC-548769 and IC-548770 were submitted along with their descriptors. These are 15 genotypes here were then procured from the gene bank for analysis of different biochemical attributes.

### Biochemical estimations

For biochemical estimations the seeds of different germplasm were properly cleaned and ground in a Willey mill to pass through a 2 mm screen and then kept in airtight bags. The crude protein content and dietary fiber were estimated by following the standard procedure given by AOAC (20). The approach described by Clegg (21) was used to estimate the total carbohydrate content.

### Estimation of ascorbic acid and niacin

Ascorbic acid and niacin content were extracted and estimated as per the method described by Sadasivam and Manickam (22). For the extraction of ascorbic acid, 3 g air-dried powdered sample was ground with 25 mL of 4% oxalic acid and filtered. Bromine water was added drop by drop to 10 mL of the filtrate until it turned orange-yellow. The excess of bromine was expelled by blowing in air. The filtrate was made up to 25 mL with 4% oxalic acid and used for ascorbic acid estimation. 2 mL of the extract was made up to 3 mL with distilled water in a test tube after that 1 mL of 2% 2, 4-dinitrophenyl hydrazine reagent and a few drops of thiourea were added with proper mixing. After 3 h incubation at 37°C, 7 mL of 80% H_2_SO_4_ was added to dissolve the osazone crystals and the absorbance was measured at 540 nm against a reagent blank. From the standard curve ascorbic acid was expressed as mg/100 g of seed flour.

For estimation of niacin content, 5 g air-dried powdered sample was steamed with 30 mL concentrated H_2_SO_4_ for 30 min. After cooling, the suspension was made up to 50 mL with distilled water and filtered. 5 mL of 60% basic lead acetate was added to 25 mL of the filtrate so obtained. After pH adjustment to 9.5 followed by centrifugation, supernatant was collected. 2 mL of concentrated H_2_SO_4_ was added to the supernatant. The mixture was allowed to stand till 1 h and centrifuged again. The 5 mL of 40% ZnSO_4_ was added to the supernatant. The pH was adjusted to 8.4 and centrifuged again. The pH of the collected supernatant was again adjusted to 7 and used as the niacin extract. For estimation, 1 mL extract was made up to 6 mL with distilled water in a test tube and 3 mL cyanogen bromide, followed by 1 mL of 4% aniline was added with shaking. The yellow colour developed after 5 min measured at 420 nm to a reagent blank. From the standard curve niacin level was expressed as mg/100 g of seed flour.

### Minerals analysis

Rice bean seed sample (500 mg) was digested with a mixture of 10 mL concentrated nitric acid, 4 mL of 60% perchloric acid and 1 mL of concentrated sulfuric acid. The digested material was diluted with 50 mL of deionized distilled water and filtered through Whatman No. 42. The volume was made up to 100 mL in a glass volumetric flask with distilled water. The minerals were analyzed by an atomic absorption spectrophotometer Shimadzu, Japan (23), whereas, the phosphorus content in the tri-acids digested extract was determined colorimetrically by method given by Dickman and Bray (24).

### Protein fractionation

The total protein (true protein) was first extracted by the method given by Bashaet al. (25). Ethanol treatment was omitted to save prolamin fraction. The extracted proteins were purified by precipitation with cold 20% trichloroacetic acid (TCA) and estimated by the method of Lowry et al. (26). The albumin and globulin fractions of the seed protein were extracted and separated according to the method of Murray (27). The prolamin fraction was extracted from the residual pellet by treating the pellet with 80% ethanol (1:10 w/v) overnight. After centrifugation at 20,000 g for 20 min at room temperature, the supernatant with prolamins was air-dried and dissolved in 0.1 N NaOH. The resulting pellet was extracted with 0.4 N NaOH (1:10 w/v) overnight and centrifuged as explained above. The supernatant was designated as glutelins. All four fractions so obtained were again precipitated and washed with cold 10% TCA and were re-dissolved in 0.2 M NaOH. The protein content was further determined by method of Lowry et al. (26).

### *In vitro* protein digestibility (IVPD)

Multi-enzyme assay was used for measuring IVPD by the method given by Hsu et al. (28). The IVPD was determined by the sequential digestion of the samples containing protein with a multi-enzyme mixture (trypsin, α-chymotrypsin and peptidase) at 37°C followed by protease at 55°C. The pH drop of the samples from pH 8.0 was recorded after 20 min of incubation. The IVPD was calculated according to the regression equation *Y* = 234.84–22.56 *X*, where *Y* is the % digestibility and *X* the pH drop.

### Fatty acid analysis

The lipids were extracted from the seeds using chloroform and methanol mixture in ratio of 2: 1 (v/v) by the method given by Folch et al. (29). Methyl esters were prepared according to the method of Metcalfe et al. (30). Fatty acid analysis was performed by gas chromatography (Shimadzu, Japan). The Peaks were identified by comparison with standards and quantified by peak area integration and expressed as weight percentage of total methyl esters; the relative weight percentage of each fatty acid was determined from integrated peak areas.

### Raffinose family oligosaccharides (RFOs)

The oligosaccharides such as raffinose, stachyose and verbascose were estimated using method given by Somiari and Balogh (31).Thin layer chromatography was applied for elution of oligosaccharides further subjected for quantitative estimation by absorbance at 432 nm in UV spectrophotometer (32).

### Estimation of anti-nutritional factors

The phenols were estimated according to the method described by Julkunen-Tiitto (33). The tannin content was estimated by the method given by Makkar et al. (34, 35). Tannic acid was used as the standard for the estimations of phenolics. Saponins and phytic acid content was determined by method given by Monago and Akhide (36). The precipitated saponins were filtered on Whatman filter paper and finally the saponinswas calculated by subtracting the weight of filter paper from the weight of filter paper along with the precipitates as:


Saponin content%=Weight ofdryfilter paper with precipitates−weight of filter paperWeight of sampleg×100

Phytic acid content was estimated by colorimetric method given by Wheeler and Ferrel (37) using ferric nitrate solution as standard and the colour was read at 480 nm. μgiron present in the test was calculated from the standard curve and phytate P was calculated as per the equation:


$$\mathrm{PhytatePmg}/g\;\mathrm{sample}=\frac{\mu g\;Fe\times 15}{\mathrm{Weight of sample}\;\left(g\right)}$$

### Antioxidant activities

Radical scavenging activity was determined using the 2,2-diphenyl-1-picryl-hydrazylhydrate (DPPH) according to method given by Diñeiro Garcíaet al. (38). 40 μL of either appropriately diluted extract or methanol in the case of the reagent blank, were added to 1.460 mL of DPPH solution (1 × 10^−4^ M) in methanol. Samples were diluted with methanol to ensure that the readings were in the linear range of the standard curve. Absorbance at 515 nm was measured after 2 h when the reaction reached its stable state. The inhibition percentage (IP) was calculated as follows:


$$\%\mathrm{IP}=\frac{A_{\mathrm{blank}}-A_{\mathrm{sample}}}{A_{blank}}\times 100$$

Where A_sample_ is the absorbance of the solution in its stable state and A_blank_ is the absorbance of DPPH solution when methanol is added rather than the sample. Trolox solutions were used to construct a standard curve and the results were expressed as μmol trolox equivalent (TE)/g.

### Flavonoid content

Total flavonoids were determined according to the methods of Nabaviet al. (39). Sample (1 g) was mixed with 10 mL 80% methanol with shaking for 2 h. Flavonoids extract (0.4 mL) was added to 4 mL of water followed by addition of 0.3 mL of 5% NaNO_2_. After 5 min, 0.3 mL of 10% AlCl_3_followed by 2 mL of 1 M NaOH after 6 min was added and the total volume was made up to 10 mL with distilled water. The color was measured at 510 nm against a blank reagent. Catechin was used as standard compound.

### α-Glucosidase inhibition

The AGI activity was determined by method given by Yao et al. (40) with slight modifications. The AGI was estimated using 50 μL of extracts with varying concentrations and incubated with 100 μL of 0.1 M phosphate buffer (pH 7.0) in 96-well plates at 37°C for 10 min. After pre-incubation, 50μLof 5 mM p-nitrophenyl-α-d glucopyranoside solution in 0.1 M phosphate buffer (pH 7.0) was added to each well at varying time intervals. The reaction mixtures were incubated at 37°C for 5 min. The absorbance was recorded at 490 nm on a microplate reader before and after incubation. The IC-50 value was defined as the concentration of bean extracts (acarbose) required to inhibit 50% of the enzyme activity. The results were expressed as a percent of AGI and calculated according to the following equation:


$$\%\mathrm{inhibition}=\frac{A_{\mathrm{control}}-A_{\mathrm{extract}}}{A_{control}}\times 100$$

Trypsin inhibitory (TI) activity was estimated by method given by Chitra and Sadasivam (41). The TI activity is expressed in trypsin inhibitory units (TIU) per mgprotein using following equation:


TIUmg/gof defatted samples=Differential Absorption×Dilution factor0.019×1000

Lipoxygenase activity was investigated by the method given by Axelrod et al. (42). One unit of lipoxygenase activity was defined as the increase in absorbance of 0.001 at 234 nm/min/mg of protein under assay conditions.

### Statistical analysis

In the present study, data were recorded in triplicates and further analyzed by one factor analysis of variance (ANOVA). When significant effects were detected by ANOVA, treatment means were compared using Duncan’s multiple range test (DMRT) at 5% of level of significant (*p* < 0.05). All statistical analyses for assessment of nutritional quality parameters of rice bean genotypes were performed with OPSTAT software (43). A correlation analysis was performed in R software at the 5% of level of significance.

## Results and discussion

**Figure 1:**
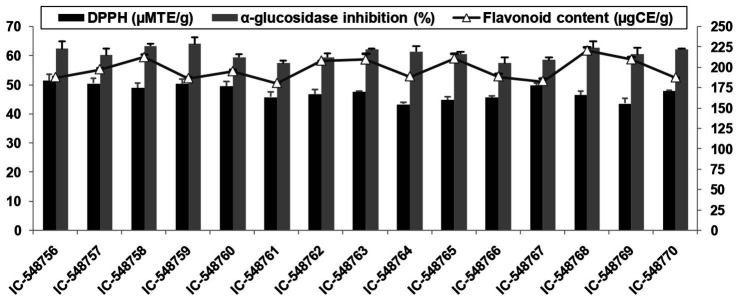
DPPH activity, AGI activity and flavonoids in rice bean genotypes (error bars represent standard deviation).

**Figure 2:**
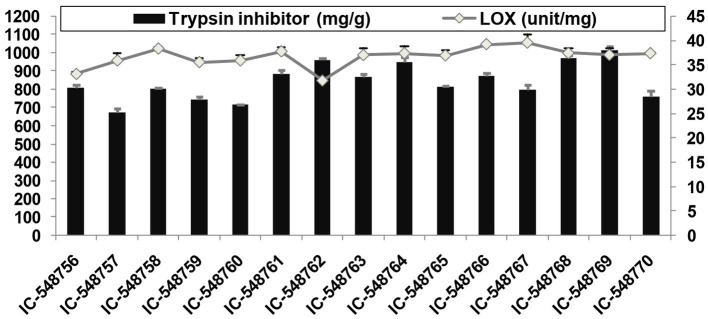
Lipoxygenase and trypsin inhibitor activities in rice bean genotypes. (Error bars represent standard deviation).

The biochemical composition, protein fractions, *in vitro* protein digestibility, minerals, essential fatty acids, anti-nutrients and antioxidant of 15 diverse rice bean genotypes has been discussed below. The nutritional attributes revealed a significant difference among genotypes (Tables 1–5; Figures 1, 2). DMRT analysis was used to determine multiple comparisons (*p* < 0.05) between genotypes.

**Table 1:** Variation in nutritional constituents in the seeds of different rice bean genotypes.

Genotypes	Crude Protein %	Total Carbohydrate (%)	Dietary fiber (%)	Lipid (%)	Ascorbic acid (mg/100 g)	Niacin (mg/100 g)
IC-548756	23.87 ± 0.06^ef^	54.47 ± 1.92^abcd^	5.13 ± 0.18^cd^	2.87 ± 0.04^d^	20.22 ± 0.28^cd^	3.56 ± 0.05^b^
IC-548757	24.92 ± 0^abcde^	52.12 ± 0.98^de^	4.98 ± 0.12^cd^	2.23 ± 0.05^h^	23.46 ± 0.44^a^	2.27 ± 0.09^e^
IC-548758	25.77 ± 0.51^abc^	56.87 ± 0.31^a^	5.25 ± 0.03^bc^	3.07 ± 0.04^bc^	18.45 ± 0.8^f^	3.05 ± 0.05^c^
IC-548759	24.87 ± 1.08^bcde^	50.56 ± 2.24^e^	4.87 ± 0.04^d^	2.77 ± 0.07^e^	17.52 ± 0.24^g^	2.87 ± 0.05^d^
IC-548760	22.56 ± 0.63^g^	55.89 ± 2.22^ab^	5.11 ± 0.23^cd^	1.98 ± 0.03^i^	21.46 ± 0.47^b^	3.87 ± 0.08^a^
IC-548761	25.44 ± 0.21^abcd^	53.16 ± 1.63^bcde^	5.87 ± 0.17^a^	1.88 ± 0.01^j^	16.56 ± 0.63^g^	3.45 ± 0.14^b^
IC-548762	23.11 ± 0.6^fg^	54.12 ± 2.39^abcd^	5.46 ± 0.21^b^	2.99 ± 0.04^c^	20.52 ± 0.89^c^	2.87 ± 0.03^d^
IC-548763	24.58 ± 0.82^de^	56.23 ± 1.37^ab^	4.98 ± 0.16^cd^	2.54 ± 0.04^f^	19.57 ± 0.05^de^	3.54 ± 0.14^b^
IC-548764	22.67 ± 0.76^g^	54.58 ± 0.5^abcd^	5.12 ± 0.03^cd^	3.17 ± 0.11^a^	18.56 ± 0.5^f^	2.25 ± 0.02^e^
IC-548765	25.87 ± 0.82^ab^	52.4 ± 2.32^cde^	5.88 ± 0.25^a^	2.42 ± 0.04^g^	18.97 ± 0.46^ef^	2.89 ± 0.03^d^
IC-548766	25.97 ± 0.61^a^	55.53 ± 0.7^abc^	5.47 ± 0.22^b^	2.88 ± 0.06^d^	17.52 ± 0.19^g^	2.74 ± 0.06^d^
IC-548767	23.54 ± 0.22^fg^	56.87 ± 2.1^a^	4.98 ± 0.22^cd^	1.87 ± 0.07^j^	20.78 ± 0.77^bc^	3.54 ± 0.14^b^
IC-548768	24.78 ± 0.47^cde^	55.41 ± 1.4^abc^	4.11 ± 0.06^f^	2.68 ± 0.03^e^	19.54 ± 0.18^de^	3.54 ± 0.1^b^
IC-548769	23.11 ± 0.3^fg^	54.92 ± 0.78^abcd^	4.56 ± 0.08^e^	2.77 ± 0.11^e^	20.25 ± 0.76^cd^	2.87 ± 0.12^d^
IC-548770	22.65 ± 0.08^g^	55.47 ± 2.3^abc^	4.97 ± 0.07^cd^	3.1 ± 0.04^ab^	18.7 ± 0.33^ef^	3.47 ± 0.03^b^

Values are means ± standard deviation of triplicates.Values with different letters in the same column are significantly different (*p* < 0.05).

**Table 2:** Variation in mineral composition in the seeds of different rice bean genotypes (mg/100 g seed flour).

Genotypes	Sodium	Potassium	Calcium	Magnesium	Phosphorous	Zinc	Copper	Iron	Manganese
IC-548756	254.87 ± 3.51^h^	1352.45 ± 47.66^cde^	387.12 ± 3.8^e^	258.42 ± 1.98^d^	412.62 ± 17.33^eg^	2.88 ± 0.03^cd^	3.71 ± 0.05^cde^	6.56 ± 0.02^e^	5.85 ± 0.07^a^
IC-548757	288.22 ± 0.26^cde^	1286.58 ± 15.08^e^	356.89 ± 9.33^f^	312.75 ± 5.07^ab^	423.44 ± 0^defg^	2.63 ± 0.06^e^	4.13 ± 0.18^b^	6.88 ± 0.2^cd^	4.88 ± 0.06^de^
IC-548758	309.45 ± 3.62^b^	1388.47 ± 1.11^bcd^	412.85 ± 3.35^d^	307.25 ± 10.53^b^	388.78 ± 12.62^hi^	3.13 ± 0.07^b^	4.56 ± 0.14^a^	5.98 ± 0.03^f^	5.23 ± 0.07^c^
IC-548759	325.54 ± 5.58^a^	1356.58 ± 15.91^bcde^	456.89 ± 10.71^c^	289.47 ± 7.57^c^	390.11 ± 11.6^hi^	3.56 ± 0.12^a^	3.23 ± 0.05^f^	6.74 ± 0.18^cde^	5.55 ± 0.16^b^
IC-548760	277.85 ± 10.27^ef^	1369.58 ± 17.27^bcd^	451.13 ± 17.89^c^	260.11 ± 9.61^d^	369.57 ± 7.33^ij^	2.99 ± 0.03^c^	3.87 ± 0.17^c^	5.99 ± 0.04^f^	4.99 ± 0.22^d^
IC-548761	263.58 ± 3.8^gh^	1410.22 ± 38.13^abcd^	414.89 ± 14.96^d^	275.65 ± 5.71^c^	352.78 ± 6.68^j^	2.87 ± 0.03^cd^	4.56 ± 0.18^a^	7.11 ± 0.03^b^	5.52 ± 0.02^b^
IC-548762	269.56 ± 7.78^fg^	1403.25 ± 3.82^abcd^	457.85 ± 5.78^c^	315.25 ± 10.8^ab^	406.62 ± 5.5^gh^	2.45 ± 0.07^f^	3.87 ± 0.09^c^	6.65 ± 0.28^de^	4.87 ± 0.07^de^
IC-548763	301.12 ± 2.44^bc^	1398.88 ± 54.22^abcd^	390.22 ± 0.4^e^	326.87 ± 0.89^a^	456.78 ± 9.88^bc^	3.11 ± 0.1^b^	3.19 ± 0.05^f^	6.95 ± 0.03^bc^	5.96 ± 0.14^a^
IC-548764	288.47 ± 3.9^cde^	1425.55 ± 26.99^ab^	370.25 ± 10.35^ef^	314.17 ± 2.83^ab^	474.52 ± 2.14^ab^	3.56 ± 0.07^a^	3.56 ± 0.14^e^	6.74 ± 0.14^cde^	4.98 ± 0.09^de^
IC-548765	298.74 ± 9.96^bcd^	1463.23 ± 59.35^a^	388.45 ± 3.85^e^	290.75 ± 1.84^c^	460.23 ± 18.25^bc^	2.98 ± 0.05^c^	3.31 ± 0.07^f^	7.14 ± 0.06^b^	4.88 ± 0.04^de^
IC-548766	307.45 ± 11.92^b^	1288.45 ± 25.55^e^	455.5 ± 18.88^c^	288.92 ± 11.46^c^	434.58 ± 17.63^de^	2.56 ± 0.1^ef^	3.87 ± 0.02^c^	7.88 ± 0.14^a^	5.54 ± 0.14^b^
IC-548767	286.54 ± 4.9^de^	1348.63 ± 59.56^de^	470.23 ± 6.36^bc^	290.17 ± 11.77^c^	434.18 ± 8.22^def^	2.88 ± 0.09^cd^	3.74 ± 0.13^cde^	6.54 ± 0.13^e^	5.56 ± 0.22^b^
IC-548768	288.74 ± 11.19^cde^	1391.25 ± 57.69^bcd^	488.5 ± 4.41^ab^	288.75 ± 3.12^c^	444.8 ± 12.43^cd^	3.45 ± 0.03^a^	3.81 ± 0.03^cd^	6.87 ± 0.09^cd^	4.87 ± 0.17^de^
IC-548769	296.87 ± 11.72^bcd^	1345.58 ± 2.24^de^	491.58 ± 19.23^a^	287.42 ± 12.83^c^	470.58 ± 11.16^ab^	2.47 ± 0.04^f^	3.61 ± 0.02^de^	7.14 ± 0.12^b^	4.74 ± 0.05^e^
IC-548770	310.54 ± 5.6^b^	1422.25 ± 30.76^abc^	460.58 ± 19.52^c^	315.54 ± 12.23^ab^	488.78 ± 21.15^a^	2.77 ± 0.1^d^	3.85 ± 0.16^c^	7.87 ± 0.13^a^	5.36 ± 0.03^bc^

Values are means ± standard deviation of triplicates. Values with different letters in the same column are significantly different (*p* < 0.05).

**Table 3:** Variation in protein fractions and *in vitro* protein digestibility in seeds of different rice bean genotypes.

Genotypes	Albumins (%)	Globulins (%)	Prolamins (%)	Glutelins (%)	*in vitro* protein digestibility (%)
IC-548756	6.5 ± 0.26^e^	11.88 ± 0.23^cde^	1.81 ± 0.09^cd^	2.44 ± 0.06^ab^	55.52 ± 0.28^b^
IC-548757	6.44 ± 0.07^e^	12.1 ± 0.41^bcde^	1.97 ± 0.08^a^	2.54 ± 0.02^a^	56.45 ± 0.31^ab^
IC-548758	6.54 ± 0.03^de^	11.69 ± 0.29^de^	1.54 ± 0.04^hi^	2.42 ± 0.05^b^	54.22 ± 0.73^c^
IC-548759	7.1 ± 0.27^bc^	11.55 ± 0.45^e^	1.85 ± 0.03^c^	2.36 ± 0.03^b^	56.87 ± 0.28^a^
IC-548760	6.85 ± 0.05^cd^	11.87 ± 0.3^cde^	1.74 ± 0.06^de^	2.45 ± 0.04^ab^	53.42 ± 0.36^c^
IC-548761	6.63 ± 0.03^de^	12.23 ± 0.41^abcd^	1.56 ± 0.02^h^	1.99 ± 0.06^e^	56.88 ± 0.31^a^
IC-548762	6.87 ± 0.16^bcd^	12.45 ± 0.28^abc^	1.96 ± 0.02^ab^	1.87 ± 0.05^f^	57.12 ± 0.3^a^
IC-548763	7.2 ± 0.24^ab^	11.65 ± 0.07^de^	1.58 ± 0.05^gh^	2.44 ± 0.06^ab^	56.23 ± 0.35^ab^
IC-548764	7.45 ± 0.04^a^	11.9 ± 0.25^cde^	1.87 ± 0.05^bc^	2.09 ± 0.05^d^	54.44 ± 0.29^c^
IC-548765	6.47 ± 0.19^e^	12.58 ± 0.22^ab^	1.74 ± 0.07^de^	1.87 ± 0.03^f^	56.98 ± 0.43^a^
IC-548766	5.88 ± 0.26^f^	11.65 ± 0.41^de^	1.67 ± 0.07^efg^	1.77 ± 0.06^f^	53.69 ± 0.87^c^
IC-548767	6.12 ± 0.08^f^	11.96 ± 0.26^cde^	1.88 ± 0.08^abc^	2.46 ± 0.05^ab^	55.45 ± 1.3^b^
IC-548768	5.83 ± 0.19^f^	12.23 ± 0.24^abcd^	1.44 ± 0.04^i^	2.53 ± 0.11^a^	55.62 ± 0.1^b^
IC-548769	6.55 ± 0.24^de^	12.55 ± 0.06^ab^	1.7 ± 0.07^ef^	1.98 ± 0.07^e^	53.63 ± 0.03^c^
IC-548770	6.88 ± 0.29^bcd^	12.72 ± 0.46^a^	1.63 ± 0.03^fgh^	2.22 ± 0.02^c^	56.78 ± 1.23^a^

Values are means ± standard deviation of triplicates.Values with different letters in the same column are significantly different (*p* < 0.05).

**Table 4:** Fatty acid profile (%) of seed lipid of different rice bean genotypes.

Genotypes	Palmitic acid (C16:0)	Stearic acid (C18:0)	Oleic acid (C18:1)	Linoleic acid (C18:2)	Linolenic acid (C18:3)
IC-548756	14.52 ± 0.47^c^	4.56 ± 0.13^g^	13.96 ± 0.13^f^	17.78 ± 0.45^ab^	35.58 ± 0.23^bcde^
IC-548757	14.92 ± 0.07^abc^	4.25 ± 0.18^h^	14.42 ± 0.29^ef^	17.12 ± 0.71^abc^	38.54 ± 1.43^a^
IC-548758	15.23 ± 0.29^abc^	4.63 ± 0.16^fg^	14.89 ± 0.51^bcde^	16.97 ± 0.52^abc^	36.55 ± 0.92^bcd^
IC-548759	14.98 ± 0.39^abc^	5.12 ± 0.12^de^	15.52 ± 0.63^abc^	17.56 ± 0.38^ab^	36.44 ± 1.51^bcd^
IC-548760	15.56 ± 0.63^a^	4.88 ± 0.14^ef^	14.87 ± 0.66^bcde^	17.88 ± 0.02^a^	34.58 ± 0.06^defg^
IC-548761	15.42 ± 0.25^a^	5.17 ± 0.2^d^	14.78 ± 0.62^cde^	16.55 ± 0^c^	34.87 ± 0.88^cdef^
IC-548762	15.36 ± 0.14^ab^	4.98 ± 0.12^de^	15.23 ± 0.19^abcd^	16.87 ± 0.62^bc^	33.56 ± 1.42^fg^
IC-548763	15.23 ± 0.27^abc^	4.56 ± 0.05^g^	14.96 ± 0.05^bcde^	17.45 ± 0.41^abc^	35.92 ± 1.23^bcde^
IC-548764	14.52 ± 0.32^c^	4.63 ± 0.21^fg^	15.56 ± 0.24^ab^	17.56 ± 0.67^ab^	34.44 ± 0.56^efg^
IC-548765	14.56 ± 0.64^bc^	5.87 ± 0.18^ab^	14.88 ± 0.31^bcde^	16.58 ± 0.68^c^	32.56 ± 1.05^g^
IC-548766	14.89 ± 0.36^abc^	5.97 ± 0.14^a^	15.55 ± 0.03^ab^	16.88 ± 0.64^bc^	34.74 ± 0.44^def^
IC-548767	15.03 ± 0.1^abc^	4.88 ± 0.11^ef^	15.85 ± 0.23^a^	17.88 ± 0.29^a^	35.71 ± 1.23^bcde^
IC-548768	15.23 ± 0.63^abc^	5.63 ± 0.15^bc^	13.98 ± 0.6^f^	16.55 ± 0.58^c^	35.52 ± 1.51^bcde^
IC-548769	14.87 ± 0.62^abc^	5.58 ± 0.18^c^	14.58 ± 0.33^def^	17.47 ± 0.27^abc^	36.78 ± 0.18^abc^
IC-548770	14.45 ± 0.29^c^	5.87 ± 0.14^ab^	15.58 ± 0.14^ab^	17.87 ± 0.14^a^	36.87 ± 1^ab^

Values are means ± standard deviation of triplicates. The absence of common letters in the same column indicates significant difference (*p* < 0.05).

**Table 5:** Variation in anti-nutritional components in seeds of different rice bean genotypes.

Genotypes	Oligosaccharide content (%)			Total phenol (%)	Simple phenols (%)	Total tannins (%)	Condensed tannins (%)	Hydrolysable tannins (%)	Phytic acid (mg/100 g)	Saponins (mg/100 g)
	Raffinose	Stachyose	Verbascose							
IC-548756	1.88 ± 0.07^ef^	1.12 ± 0.03^g^	1.12 ± 0.03^b^	1.98 ± 0.03^a^	0.55 ± 0.04^b^	1.44 ± 0.04^a^	0.74 ± 0.03^def^	0.7 ± 0.04^ab^	3.67 ± 0.07^e^	1.2 ± 0.04^b^
IC-548757	2.23 ± 0.04^bc^	0.94 ± 0.03^h^	0.98 ± 0.03^c^	1.66 ± 0.05^e^	0.45 ± 0.04^c^	1.21 ± 0.05^f^	0.66 ± 0.03^gh^	0.55 ± 0.03^f^	3.22 ± 0.05^f^	1.23 ± 0.04^b^
IC-548758	2.14 ± 0.06^c^	1.54 ± 0.06^a^	0.87 ± 0.04^d^	1.56 ± 0.05^f^	0.56 ± 0.03^b^	1 ± 0.05^h^	0.67 ± 0.03^gh^	0.33 ± 0.04^h^	3.56 ± 0.04^e^	1.44 ± 0.04^a^
IC-548759	1.99 ± 0.05^d^	1.45 ± 0.06^b^	1.23 ± 0.04^a^	1.88 ± 0.05^bc^	0.44 ± 0.03^c^	1.44 ± 0.03^ab^	0.84 ± 0.04^a^	0.6 ± 0.02^def^	4.12 ± 0.12^ab^	0.96 ± 0.03^d^
IC-548760	1.85 ± 0.04^f^	0.97 ± 0.04^h^	0.88 ± 0.02^d^	1.63 ± 0.03^ef^	0.58 ± 0.03^b^	1.05 ± 0.04^gh^	0.56 ± 0.02^i^	0.49 ± 0.03^g^	4.11 ± 0.12^ab^	1.11 ± 0.06^c^
IC-548761	2.25 ± 0.05^b^	1.12 ± 0.02^g^	1.09 ± 0.02^b^	1.87 ± 0.03^c^	0.47 ± 0.03^c^	1.4 ± 0.05^abcde^	0.69 ± 0.06^fgh^	0.71 ± 0.03^a^	3.87 ± 0.12^cd^	1.23 ± 0.02^b^
IC-548762	2.56 ± 0.05^a^	1.23 ± 0.04^f^	0.99 ± 0.04^c^	1.78 ± 0.03^d^	0.68 ± 0.03^a^	1.1 ± 0.03^g^	0.77 ± 0.03^bcd^	0.33 ± 0.04^h^	3.66 ± 0.14^e^	1.07 ± 0.03^c^
IC-548763	1.87 ± 0.05^ef^	1.45 ± 0.06^b^	1.1 ± 0.04^b^	1.96 ± 0.05^ab^	0.59 ± 0.02^b^	1.37 ± 0.02^de^	0.8 ± 0.03^abc^	0.57 ± 0.05^ef^	3.99 ± 0.09^abc^	0.87 ± 0.05^e^
IC-548764	2.24 ± 0.06^bc^	1.56 ± 0.03^a^	1.23 ± 0.05^a^	1.87 ± 0.07^c^	0.46 ± 0.03^c^	1.41 ± 0.04^abcde^	0.76 ± 0.03^bcd^	0.65 ± 0.02^bcd^	4.16 ± 0.1^a^	1.22 ± 0.03^b^
IC-548765	2.55 ± 0.03^a^	1.23 ± 0.05^f^	0.98 ± 0.03^c^	2.01 ± 0.07^a^	0.65 ± 0.05^a^	1.36 ± 0.03^e^	0.7 ± 0.03^efg^	0.66 ± 0.04^abc^	4.07 ± 0.04^ab^	1.11 ± 0.02^c^
IC-548766	1.98 ± 0.07^d^	1.35 ± 0.03^cd^	1.11 ± 0.04^b^	1.96 ± 0.05^ab^	0.58 ± 0.03^b^	1.38 ± 0.03^acde^	0.75 ± 0.02^cde^	0.63 ± 0.03^cd^	4.15 ± 0.08^ab^	1.01 ± 0.04^d^
IC-548767	1.96 ± 0.06^de^	1.42 ± 0.03^bc^	0.87 ± 0.03^d^	1.88 ± 0.05^bc^	0.44 ± 0.04^c^	1.24 ± 0.04^f^	0.64 ± 0.03^h^	0.6 ± 0.02^def^	3.97 ± 0.17^bc^	1.07 ± 0.03^c^
IC-548768	2.23 ± 0.08^bc^	1.25 ± 0.02^ef^	0.88 ± 0.03^d^	1.97 ± 0.05^a^	0.54 ± 0.04^b^	1.43 ± 0.03^abc^	0.81 ± 0.04^ab^	0.62 ± 0.03^cde^	4.07 ± 0.03^ab^	0.98 ± 0.03^d^
IC-548769	2.21 ± 0.05^bc^	1.32 ± 0.04^de^	1.02 ± 0.03^c^	1.88 ± 0.07^bc^	0.46 ± 0.03^c^	1.42 ± 0.03^abcd^	0.73 ± 0.04^def^	0.69 ± 0.04^ab^	3.88 ± 0.05^cd^	1.12 ± 0.05^c^
IC-548770	1.98 ± 0.05^d^	1.02 ± 0.04^h^	0.97 ± 0.05^c^	1.79 ± 0.05^d^	0.54 ± 0.04^b^	1.25 ± 0.03^f^	0.69 ± 0.03^fgh^	0.56 ± 0.02^f^	3.73 ± 0.05^de^	0.99 ± 0.03^d^

Values are means ± standard deviation of triplicates. Values with different letters in the same column are significantly different (*p* < 0.05).

### Nutritional compositions

Carbohydrates, protein and lipid are essential for maintaining different biological processes. Table 1 shows nutritional composition (crude protein, total carbohydrates, lipid, dietary fiber, ascorbic acid and niacin) in 15 rice bean genotypes. The crude protein, total carbohydrates and lipid content in rice bean genotypes ranged from 22.56% (IC-548760) to 25.97% (IC-548766), 50.56% (IC-548759) to 56.87% (IC-548758) and 1.87% (IC-548767) to 3.17% (IC-548764), respectively (Table 1). The variation in crude protein content from 18.08–25.57% has also been reported earlier (11, 44). Different group of researchers has reported variation in crude fiber from 11.20 to 13.0% in different *Vigna* species (11, 45–47). The major fraction of nutrition was comparable in different *Vigna* species (48). The dietary fibercontent was highest in genotype IC-548765 (5.88%). Dietary fiber observed 3.5, 1.9, and 4.2% in green frozen pea, green beans and lima bean, respectively (49). The dietary fiber content in the different genotypes under study was higher than the released rice bean varieties RBL1 and RBL6 with 3.0 and 3.5 g/100 g, respectively (44). The ascorbic acid and niacin was higher in the genotypes IC-548757 (23.46 mg/100 g) and IC-548760 (3.87 mg/100 g), respectively. The ascorbic acid content was higher than earlier reports in chickpea (50), *Vigna radiata* and *Vigna mungo* (48, 51). The niacin content in rice bean genotypes under study was higher as compared to earlier reports on *Vigna radiate* (52), pigeon pea, Indian bean, black gram (53); catjang cowpea (54), *Vigna mungo* (55) and winged bean (56). As established earlier, the present study also revealed the nutritional superiority of rice bean over most of other common pulses.

### Mineral contents

Minerals are important for different cellular and physiological functions in the body. Deficiency in minerals such as potassium, sodium, calcium, iron, and zinc is often associated with different health issues. Both macro-minerals (calcium, phosphorus, magnesium, sodium and potassium) and trace-minerals (iron, manganese, copper and zinc; Table 2) were estimated in 15 rice bean genotypes. The macro- and trace-minerals are important as cofactors in various metabolic reactions (11). The macro-minerals including sodium (87to 325.54 mg/100 g), potassium (1286.58 to 1463.23 mg/100 g), phosphorus (352.78to 488.78 mg/100 g), magnesium (258.42 to 326.87 mg/100 g) and calcium (356.89 to 491.58 mg/100 g) revealed significant variations among rice bean genotypes. The trace-minerals also revealed variation in zinc (2.45 to 3.56 mg/100 g), copper (3.19 mg/100 g to 4.56 mg/100 g), iron (5.98 to 7.88 mg/100 g) and manganese (4.74 to 5.96 mg/100 g) in different rice bean genotypes. The genotype IC-548759 was recorded with comparatively higher sodium and zinc content; genotype IC-548758 with higher zinc, copper, potassium, phosphorus, sodium content whereas genotype IC-548763 with higher magnesium and manganese content as compared to other genotypes of the group. In totality, genotype IC-548770 was observed with comparatively higher proportion of the minerals *viz.,* sodium, potassium, calcium, magnesium, phosphorous, copper, iron and manganese and could be considered for biofortification programmes. The mineral content in rice bean was comparable or even higher than those reported in other legumes such as common bean, pea, lentil, cowpea and chickpea (57). Copper, manganese, iron and zinc levels in the common beans with mean value of 0.6 mg/100 g, 2.5 mg/100 g, 4.6 mg/100 g, 2.8 g/100 g, respectively (58).

### Protein fractions and *in vitro* protein digestibility (IVPD)

Protein fractions (albumins, prolamins, glutelins and globulins) and *in vitro* protein digestibility of rice bean genotypes has been presented in Table 3. The legumes have higher protein than cereals and considered as affordable source of protein and nutrients for weaker sections of society. In present study, among genotypes significant difference was observed in different protein fractions and globulins dominated over the protein fractions. Globulins had higher proportion among all the protein fractions (11). Different protein fractions in rice bean revealed globulins (11.55 to 12.72%) in the highest proportion followed by albumins (5.83 to 7.45%), glutelins (1.77 to 2.54%) and prolamins (1.44 to 1.97%). In present study, rice bean genotypes had 20.59–24.68% extractable proteins from seed flour. In other study, winged beans have been reported with 26.46% albumin, 31.94% globulin, 12.59% glutelin and 4.05% prolamin, making a total 75.04% extractable proteins from the defatted flour (59, 60). The consumption of enough protein does not give a guarantee of fulfilling the requirements of the amino acids, but the digestibility of available proteins is also a crucial factor for determining protein quality (61). Rice bean has highly digestible proteins with the digestibility range from 53.42 to 57.12%. The highest IVPD was recorded in the genotype IC-548762 while the least value was observed in genotype IC-548760. Jood and others observed significant variations in IVPD of chickpea and black gram varieties from 48 to 53% and 52 to 58%, respectively (62).

### Fatty acid fractions

Table 4 reveals fatty acid profile (palmitic acid, stearic acid, oleic acid, linoleic acid and linolenic acid) in 15 rice bean genotypes. Although, lipid content is lesser in rice bean as compared to many other legumes of *Vigna* family, but the fatty acids composition is superior with low levels of saturated fatty acids and higher content of unsaturated fatty acids. Unsaturated fatty acids are nutritionally desirable for different metabolic functions of the body (11, 63). Furthermore, PUFAs were in higher proportion as compared to monounsaturated fatty acids (MUFAs). The linolenic acid content was highest (32.56 to 38.54%) followed by linoleic acid (16.55 to 17.88%), oleic acid (13.96 to 15.85%), palmitic acid (14.45 to 15.56%) and steric acid (4.25 to 5.97%). The highest level of linolenic acid was observed in genotype IC-548757 (38.54%) followed by IC-548770. Moth bean lipid fractions revealed palmitic acid, linolenic acid and linoleic acid as major fatty acids (64) however, the proportions of linolenic acid were considerably low in comparison to presently investigated rice bean genotypes. The fatty acids profile of rice bean, adzuki bean and closely related *Vigna* species have also been reported earlier (10, 65). Furthermore, in the present study, rice bean genotypes revealed higher ratio of linolenic acid: linoleic acid (*n-6/n-3* ratio) than previously reported beans, i.e., navy bean, butter bean, white bean, kidney bean and black bean (66).

### Anti-nutritional factors

The digestibility in legumes is commonly affected by anti-nutritional factors such as phytate, RFOs, tannins, phenolics, trypsin inhibitors and saponins (65). These anti-nutrients in legumes generally affectthe absorption of available nutrients thereby decrease their culinary value. The most commonly anti-nutritional factors present in pulses are phenolics, tannins, saponins, phytic acid and raffinose family of oligosaccharides (RFOs). RFOs collectively in legumes include raffinose (trisaccharide), staychose (tetrasaccharide) and verbascose (pentasaccharide). The levels of oligosaccharides are comparatively low in rice bean as compared to soybean (67), which is commonly associated with flatulence after consumption. Thevariation in the level of anti-nutritional factors in rice bean genotypes is presented in Table 5. The levels of RFOs *viz.*, raffinose, styachose and verbascose in rice bean genotypes ranged from 1.85 to 2.56%, 0.94 to 1.56% and 0.87 to 1.23%, respectively (Table 5).Verbascose (3.32%) in raw faba beans and stachyose in the remaining legumes (2.21–3.23%) has been reported as main oligosaccharide associated with flatulence (68).The total oligosaccharide content in raw legumes was reported 70.7 mg/g in yellow peas and 144.9 mg/mg/g in chickpeas (69).

The phytic acid in legumes affects absorption of minerals including iron, zinc, magnesium,calciumetc. Phytic acid and saponinsrevealed a significant variation among rice beangenotypes. The values for phytic acid varied from 3.22 mg/100 g (IC-548757) to 4.16 mg/100 g (IC-548764) with a mean value 3.88 mg/100 g. It is also known to form complexes with protein and carbohydrates and affect their digestion and absorption (70). The presently investigated genotypes revealed lesserphytic acid content as compared to commonly consumed legumes including black gram (71); lablab bean (72); tribal pulses velvet-bean (73), purple mucuna (74) And*vigna mungo* (75).

Phenolic compounds are known to inhibit the activity of digestive protein and enzymes like α-amylase, trypsin, chymotrypsin and lipase besides decreasing the digestibility of proteins, carbohydrates and availability of vitamins and minerals (76).Total phenol and simple phenolics in rice bean genotypes ranged from 1.56 to 2.01% and 0.44 to 0.68%, respectively. Minimum values for total and simple phenolics were reported in genotype IC-548758 and IC-548767, respectively. Katoch and other workers have earlier reported nearly similar range of phenolics content in rice bean genotypes (11, 77–79).

Saponin interferes with absorption of normal nutrients and minerals. These are not readily hydrolyzed by the human digestive enzymes, thereby impairing gastrointestinal digestion (80). Tannins are often considered as anti-nutrient as they bind and precipitate proteins and form complex bonds with starch, cellulose and iron and other minerals which affect their digestibility. In current study, saponins content in rice bean genotypes ranged from 0.87 (IC-548763) to 1.44 mg/100 g (IC-548758). The total tannins, condensed tannins and hydrolysable tannins in rice bean genotypes ranged from 1.00 to 1.44%, 0.56 to 0.84% and 0.33 to 0.71%, respectively (Table 5). The genotypes IC-548758 and IC-548760 exhibited comparatively lower value for total tannins. Overall, in the study revealed that the superiority of rice bean genotypes *viz.*, IC-548760, IC-548757 and IC-548770 with comparatively lower levels of antinutrients. Conversely, the higher proportion of antinutrients was recorded in genotype IC-548764.In another study, saponin content 1.2 mg/100 g and 2.5 mg/100 g has been reported in rice bean genotypes IC-137194and JCR-163, respectively (11).The phytate and saponin contents in raw *Vigna racemosa* were reported to the extent of 0.31, 0.87%, respectively (81). Saponins content ranging from 4.73 to 17.98 mg/g have been reported in pigeonpea genotypes (82). In one of the study the tannins content in rice bean genotypes has been reported in the range of 1.2 to1.5% while in other legumes from 1.15 to 1.96% (11, 47).

### Antioxidants activities

The human body requires different antioxidants to prevent and overcome the effect of free radicals generated in different metabolic processes. Free radicals could adversely alter cellular molecules (nucleic acid, lipids and proteins) and mediate many diseases (cardiac disease, diabetes and cancer) in human body. Antioxidants quench free radicals or reactive oxygen species (ROS) by retarding the oxidation of cellular molecules (83). Rice bean is a good source of antioxidants such as flavonoids and DPPH. The antioxidant content in 15 rice bean genotypes are presented in Figures 1, 2. DPPH based free radical scavenging activity in rice bean genotypes ranged from 43.26 (IC-548764) to 51.52μMTE/g(IC-548756) with mean value of 47.48μMTE/g (Figure 1). The free radical scavenging activity was the highest in genotype IC-548756 among other rice bean genotypes.Rice beans revealed a significant difference in their DPPH based free radical scavenging activity ranging from 39.87–46.40μMTE/g (79).

Flavonoids act as an antioxidant and prevent diseases such as cancer, inflammation, autoimmune diseases, cataract, arteriosclerosis and aging. The flavonoids in rice bean genotypes under present study ranged from 180.46 (IC-548761) to 220.25μgCE/g (IC-548768) with an average value of 197.53μgCE/g (Figure 1). Rice beans had significant difference in their total flavonoids ranging from 55.95–320.39μgCE/g (79). Flavonoid content in pigeonpea genotypes was reported ranging from 5.32 to 16.02 mg/100 gand in kidney beans from 86.16 to 1008.69 mg RE/100 g DW (78, 82).

The AGI functions to inhibit the quick absorption of sugar in the blood (84). Recent research has shown that AGI may also be helpful as COVID-19 antivirals (85). In present study, the AGI activity ranged from 57.44% (IC-548761) to 64.14% (IC-548759) in rice bean genotypes (Figure 1). Different varieties of rice beans from China were reported withsignificant differences in their AGI activity ranging from 44.32–68.71% (79).

Lipoxygenase activity related to off-flavor and TI activity affecting protein digestibility, are among undesirable components present in rice bean affecting its culinary quality. The TI content in rice bean genotypes varied from 25.27 (IC-548757) to 37.98 mg/g (IC-548769) with an average value (31.60 mg/g; Figure 2). The TI content in seeds ranging from 27.4 to 30.6 mg/g with the highest activity in genotype BRS-2 (30.6 mg/g) (17). The genotype IC-548762 was observed with minimal TI content. The TI values for rice bean cultivars revealed significant differences between the cultivars and were in the similar range as in other legumes (47, 75). It is well known that lipoxygenase catalyzes the oxidation of polyunsaturated fatty acids to produce peroxides (86). The lipoxygenase activity in different rice bean genotypes ranged from 846.53 (IC-548762) to 1056.23unit/mg (IC-548767) with an average of 978.99unit/mg (Figure 2). Canola seeds (*Brassica napus* var. wester) and observed 7571unit/mg of lipoxygenase (87). Lipoxygenase activity in rice bean genotypes varying from 732 units/mg (BRS-2) to 820 units/mg (JCR-20) (88).

### Correlation analysis

**Figure 3:**
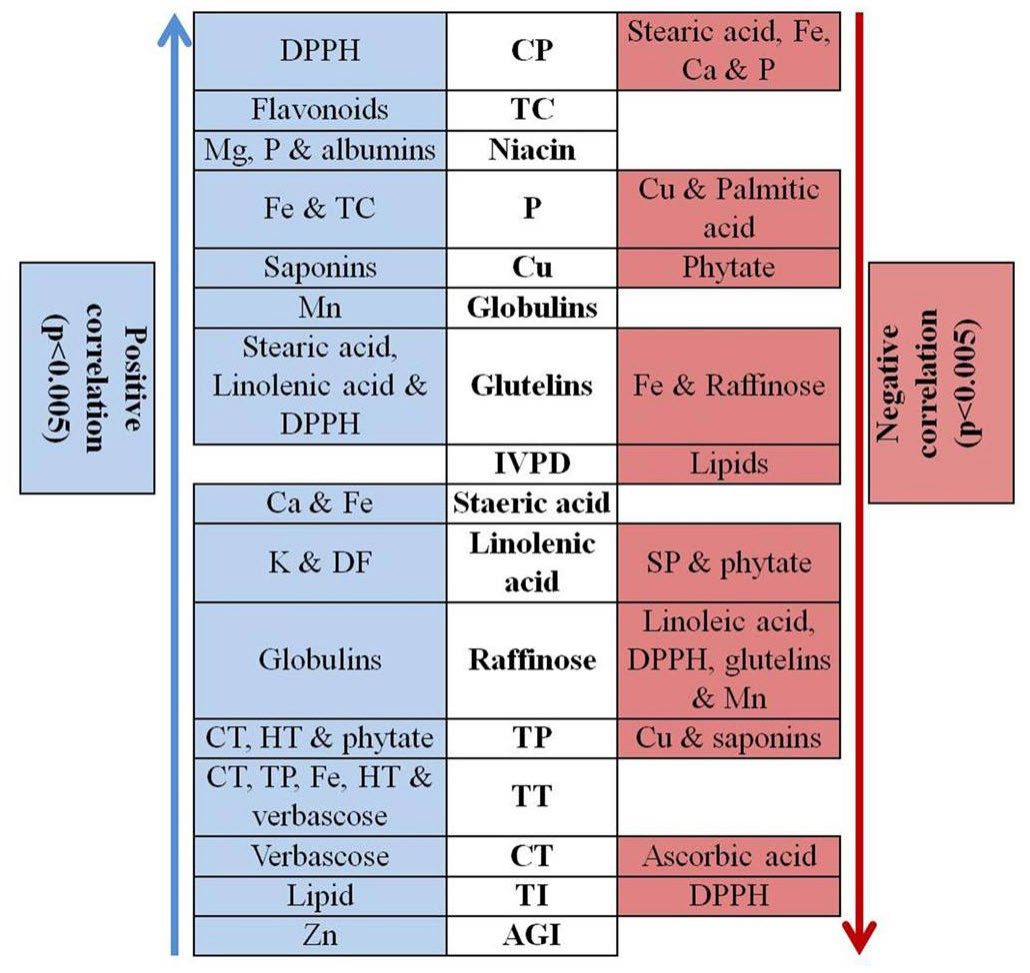
Correlation analysis of different quality traits in rice bean genotypes. Na – Sodium, K – Potassium, Ca – Calcium, Mg – Magnesium, P – Phosphorous, Zn – Zinc, Cu – Copper, Cu – Iron, Mn – Manganese, CP – Crude Protein content, TC – Total carbohydrate, DF – dietary fibre, IVPD – *in vitro* protein digestibility, CT – condensed tannin, TP – total phenol, HT – Hydrolysable tannins, TI – trypsin inhibitors, AGI-α – glucosidase inhibition, TT – total tannins.

Correlation analysis was performed among different quality parameters in rice bean (Figure 3). Micronutrients such as iron, zinc, copper, manganese and magnesium showed insignificant correlation among them whereas, zinc revealed positive association with AGI. Usually a negative correlation was reported between iron and zinc posing a significant challenge for genetic bio fortification in legumes (89). Our results suggest that the accumulation and enhancement of one type of mineral do not others and they are independently inherited in rice bean. The results are in agreement with Welch and Graham (90) who reported that different minerals exhibit independent genetic control. The crude protein content showed positive association with DPPH whereas negative correlation was observed with stearic acid, iron, calcium and phosphorous. *In vitro* protein digestibility was negatively associated with total lipids whereas total carbohydrate revealed positive correlation with flavonoids. Phosphorus content was positively correlated with iron and total carbohydrate while negative association was observed with copper and palmitic acid. Among oligosaccharides, raffinose showed positive correlation with globulins while negative correlation was observed with linolenic acid, DPPH, glutelins, and manganese. Phytic acid is especially known as a chelating agent that reduces the bioavailability of minerals especially iron, calcium and magnesium. In present study, phytic acid showed positive correlation with total phenol while negative association with linolenic acid and copper suggested that phytic acid could impair the copper bio-accessibility. In contrast, low phenol content can be directly reduced by decreasing the amount of phytic acid. There was an insignificant association between phytic acid and minerals especially iron, magnesium.

On the basis of the results of the present study, cumulative rating for the characterization of rice bean genotypes was carried out for the selection of superior genotypes. The cumulative rating revealed that genotypes IC-548760 followed by IC-548757 and IC-548770 were having lesser levels of anti-nutrients. The genotype IC-548768 revealed high AGI, flavonoids, calcium, zinc, glutelins and lower saponin while genotype IC-548756 had high free radical scavenging activity, manganese, niacin content and lower raffinose content and lipoxygenase activity. The genotype IC-548758 had high crude protein, total carbohydrates, lipid, minerals (zinc, copper, potassium, phosphorous, and sodium), AGI, flavonoids and lower verbascose, total tannin, total phenol, hydrolysable tannins and phytic acid. Based on the overall ranking, genotypes IC-548770, IC-548758, and IC-548760 revealed superiorquality attributes and normal levels of antinutrients. The study therefore highlights the potential of specific rice bean genotypes in the development of nutrient-enriched food and as functional ingredients in diets designed for mitigation of nutritious food insecurity.

## Conclusion

In the recent years global demand for nutraceutical has resulted renewed interest in underutilized legumes endowed with higher nutritional and therapeutic properties. As pulses with high yield and better nutritional quality are being identified for introduction in different parts of the world, rice bean seems to be a suitable choice for addition to the presently existing list of pulses. Rice bean legume is a rich source of nutrients including proteins, carbohydrates, fatty acid, vitamins and minerals along with anti-oxidant and anti-diabetic potential. In the present study 15 rice bean genotypes were compared for nutritional, anti-nutritional and antioxidant potential. The genotype IC-548758 revealed higher levels of nutrient fraction with lower levels of anti-nutrients whereas, genotypes IC-548757 and IC-548759 have lower levels of anti-nutrients with higher anti-oxidant activity. Based on genotypic rating genotypes IC-548770, IC-548758, and IC-548760 reflected desirable nutritional attributes with the potential in genetic biofortification and improvement programmes.It is therefore imperative to further explore the potential of rice bean legume and promote its usage for food as well as value added products which will benefit different sectors of the society including common masses, farmers and food industry.

## Data availability statement

The raw data supporting the conclusions of this article will be made available by the authors, without undue reservation.

## Author contributions

RK conceived the project and performed data collection and analysis and wrote the manuscript. All authors contributed to the article and approved the submitted version.

## Conflict of interest

The authors declare that the research was conducted in the absence of any commercial or financial relationships that could be construed as a potential conflict of interest.

## Publisher’s note

All claims expressed in this article are solely those of the authors and do not necessarily represent those of their affiliated organizations, or those of the publisher, the editors and the reviewers. Any product that may be evaluated in this article, or claim that may be made by its manufacturer, is not guaranteed or endorsed by the publisher.

## Abbreviations

AOAC: Association of Official Analytical Chemists
DMRT: Duncan’s multiple range test
DPPH: 2,2-diphenyl-1-picryl-hydrazylhydrate
FAO: Food and Agriculture Organization
IVPD: *In vitro* protein digestibility
NBPGR: National Bureau of Plant Genetic Resources, New Delhi
NUS: neglected and/or underutilized crops species
PUFAs: Polyunsaturated fatty acids
RFOs: Raffinose family oligosaccharides
WHO: World Health Organization

## References

[1] Katoch R In: Katoch R, editor. Exploiting the nutritional potential of an underutilized legume. 1st ed. Singapore: Springer (2020). 386.

[2] Vetriventhan M Upadhyaya HD Dwivedi SL Pattanashetti SK Singh SK. Finger and foxtail millets In: Singh M Upadhyaya HD, editors. Genetic and genomic resources for grain cereals improvement. New York, USA: Elsevier Inc. (2016). 291–319.

[3] Kumari A Chaudhary HK. Nutraceutical crop buckwheat: a concealed wealth in the lap of Himalayas. Crit Rev Biotechnol. (2020) 40:539–54. doi: 10.1080/07388551.2020.1747387, PMID: 32290728

[4] Lepcha P Egan AN Doyle JJ Sathyanarayana N. A review on current status and future prospects of winged bean (*Psophocarpus tetragonolobus*) in tropical agriculture. Plant Foods Hum Nutr. (2017) 72:225–35. doi: 10.1007/s11130-017-0627-0, PMID: 28866817

[5] Tanzi AS Eagleton GE Ho WK Wong QN Mayes S Massawe F. Winged bean (*Psophocarpus tetragonolobus* (L.) DC.) for food and nutritional security: synthesis of past research and future direction. Planta. (2019) 250:911–31. doi: 10.1007/s00425-019-03141-2, PMID: 30911885

[6] Mabhaudhi T Chimonyo V Modi A. Status of underutilised crops in South Africa: opportunities for developing research capacity. Sustainability. (2017) 9:1569. doi: 10.3390/su9091569

[7] Mabhaudhi T Chimonyo VGP Chibarabada TP Modi AT. Developing a roadmap for improving neglected and underutilized crops: a case study of South Africa. Front. Plant Sci. (2017) 8:2143. doi: 10.3389/fpls.2017.02143, PMID: 29312397 PMC5735103

[8] Kaul T Sony SK Bharti J Verma R Nehra M Thangaraj A. Rice bean-an underutilized food crop emerges as cornucopia of micronutrients essential for sustainable food and nutritional security In: Chapman MA, editor. Underutilised crop genomes. Cham, Switzerland: Springer (2022). 301–14.

[9] Ayilara MS Abberton M Oyatomi OA Odeyemi O Babalola OO. Potentials of underutilized legumes in food security. Front Soil Sci. (2022) 2:1–12. doi: 10.3389/fsoil.2022.102019336733849

[10] Katoch R. Morpho-physiological and nutritional characterization of ricebean (*Vigna umbellata*). Acta Agron Hungarica. (2011) 59:125–36. doi: 10.1556/AAgr.59.2011.2.3

[11] Katoch R. Nutritional potential of rice bean (*Vigna Umbellata*): an underutilized legume. J Food Sci. (2013) 78:C8–C16. doi: 10.1111/j.1750-3841.2012.02989.x, PMID: 23278402

[12] Atta K Adhikary S Mondal S Mukherjee S Pal A Mondal S. A review on stress physiology and breeding potential of an underutilized, multipurpose legume: Rice bean (*Vigna umbellata*) In: Jha UC Nayyar H Agrawal SK Siddique KHM, editors. Developing climate resilient grain and forage legumes. Singapore: Springer (2022). 235–53.

[13] Bhagmal JV. Under-utilised plant resources In: Paroda RS Arora RK, editors. Plant genetic resources conservation and management concepts and approaches. New Delhi, India: the International Board for Plant Genetic Resources (1991)

[14] Rodriguez MS Mendoza EMT. Nutritional assessment of seed proteins in rice bean *Vigna Umbellata* (thumb.) Ohwi and Ohashi. Plant Foods Hum Nutr. (1991) 41:1–9. doi: 10.1007/BF02196376 2017423

[15] Dhillon PK Tanwar B. Rice bean: a healthy and cost-effective alternative for crop and food diversity. Food Secur. (2018) 10:525–35. doi: 10.1007/s12571-018-0803-6

[16] Bhagyawant SS Bhadkaria A Narvekar DT Srivastava N. Multivariate biochemical characterization of rice bean (*Vigna umbellata*) seeds for nutritional enhancement. Biocatal Agric Biotechnol. (2019) 20:101193. doi: 10.1016/j.bcab.2019.101193

[17] Katoch R Sharma K Singh SK Thakur N. Evaluation and characterization of trypsin inhibitor from rice bean with inhibitory activity against gut proteases of *Spodoptera litura*. Z Naturforsch C J Biosci. (2015) 70:287–95. doi: 10.1515/znc-2015-5029 26618568

[18] Tripathi A Hallan V Katoch R. Cloning, characterization, expression analysis, and agglutination studies of novel gene encoding β-d-galactose, N-acetyl-d-glucosamine and lactose-binding Lectin from Rice bean (*Vigna umbellata*). Mol Biotechnol. (2022) 64:293–310. doi: 10.1007/s12033-021-00410-y, PMID: 34611825

[19] Kaul T Easwaran M Thangaraj A Meyyazhagan A Nehra M Raman NM. De novo genome assembly of rice bean (*Vigna umbellata*) – a nominated nutritionally rich future crop reveals novel insights into flowering potential, habit, and palatability centric – traits for efficient domestication. Front Plant Sci. (2022) 13:1–21. doi: 10.3389/fpls.2022.739654, PMID: 36267942 PMC9577371

[20] AOAC In: George W Latimer J, editors. Official methods of analysis of AOAC International. 20th ed. Maryland, USA: AOAC INTERNATIONAL (2016). 3172.

[21] Clegg KM. The application of the anthrone reagent to the estimation of starch in cereals. J Sci Food Agric. (1956) 7:40–4. doi: 10.1002/jsfa.2740070108

[22] Sadasivam S Manickam A In: Sadasivam S Manickam A, editors. Biochemical methods. 1st ed. New Delhi, India: New Age International Pvt. Limited (1996). 198.

[23] Isaac RA Johnson WC. Collaborative study of wet and dry ashing techniques for the elemental analysis of plant tissue by atomic absorption spectrophotometry. J Assoc Off Anal Chem. (1975) 58:436–40. doi: 10.1093/jaoac/58.3.436

[24] Dickman SR Bray RH. Colorimetric determination of phosphate. Ind Eng Chem Anal Ed. (1940) 12:665–8. doi: 10.1021/ac50151a013

[25] Basha SMM Cherry JP Young CT. Changes in free amino acids, carbohydrates, and proteins of maturing seeds from various Peanut (*Arachis hypogaea* L.) cultivars. Cereal Chem. (1976) 53:586–96.

[26] Lowry OH Rosebrough NJ Farr AL Randall RJ. Protein measurement with the Folin phenol reagent. J Biol Chem. (1951) 193:265–75. doi: 10.1016/s0021-9258(19)52451-6 14907713

[27] Murray DR. The seed protein of kowhai, *Sophora microphylla* AIT. Z Pflanzenphysiol. (1979) 93:423–8. doi: 10.1016/S0044-328X(79)80176-2

[28] Hsu HW Vavak DI Satterlee LD Miller GA. A Multienzyme Technique for Estimating Protein Digestibility. J Food Sci. (1977) 42:1269–73. doi: 10.1111/j.1365-2621.1977.tb14476.x

[29] Folch J Lees M Solane-Stanly GM. A simple method for the isolation and purification of total lipides from animal tissues. J Biol Chem. (1957) 226:497–509. doi: 10.1016/S0021-9258(18)64849-5, PMID: 13428781

[30] Metcalfe LD Schmitz AA Pelka JR. Rapid preparation of fatty acid esters from lipids for gas chromatographic analysis. Anal Chem. (1966) 38:514–5. doi: 10.1021/ac60235a044

[31] Somiari RI Balogh E. Effect of soaking, cooking and crude α-galactosidase treatment on the oligosaccharide content of cowpea flours. J Sci Food Agric. (1993) 61:339–43. doi: 10.1002/jsfa.2740610308

[32] Tanaka M Thananunkul D Lee TC Chichester CO. A simplified method for the quantitative determination of sucrose, raffinose and stachyose in legume seeds. J Food Sci. (1975) 40:1087–8. doi: 10.1111/j.1365-2621.1975.tb02274.x

[33] Julkunen-Tiitto R. Phenolic constituents in the leaves of northern willows: methods for the analysis of certain phenolics. J Agric Food Chem. (1985) 33:213–7. doi: 10.1021/jf00062a013

[34] Makkar HPS. Effects and fate of tannins in ruminant animals, adaptation to tannins, and strategies to overcome detrimental effects of feeding tannin-rich feeds. Small Rumin Res. (2003) 49:241–56. doi: 10.1016/S0921-4488(03)00142-1

[35] Makkar HPS Blümmel M Borowy NK Becker K. Gravimetric determination of tannins and their correlations with chemical and protein precipitation methods. J Sci Food Agric. (1993) 61:161–5. doi: 10.1002/jsfa.2740610205

[36] Monago CC Akhide V. Estimation of tannin, Saponin, oxalate, cyanogenic and cardiac glycosides in Garsinia Kola. J Appl Sci Environ Manag. (2002) 6:22–5. doi: 10.4314/jasem.v6i1.17189

[37] Wheeler EL Ferrel RE. A method for Phytic acid determination in wheat and wheat fractions. Cereal Chem. (1971) 48:312–20.

[38] Diñeiro García Y Suáarez Valles B Picinelli LA. Phenolic and antioxidant composition of by-products from the cider industry. Food Chem. (2009) 117:731–8. doi: 10.1016/j.foodchem.2009.04.049

[39] Nabavi SM Ebrahimzadeh MA Nabavi SF Hamidinia A Bekhradnia AR. Determination of antioxidant activity, phenol and flavonoids content of *Parrotia persica* Mey. Pharmacol. (2008) 2:560–7.

[40] Yao Y Wei S Meijing Z Ren G. Antioxidant and α-glucosidase inhibitory activity of colored grains in China. J Agric Food Chem. (2010) 58:770–4. doi: 10.1021/jf903234c, PMID: 19904935

[41] Chitra R Sadasivam S. A study of the trypsin inhibitor of black gram (*Vigna mungo* (L.) Hepper). Food Chem. (1986) 21:315–20. doi: 10.1016/0308-8146(86)90065-8

[42] Axelrod B Cheesbrough TM Laakso S. Lipoxygenase from soybeans: EC 1.13.11.12 linoleate:oxygen oxidoreductase. Methods Enzymol. (1981) 71:441–51. doi: 10.1016/0076-6879(81)71055-3

[43] Sheoran O Tonk D Kaushik L Hasija RC Pannu RS. Statistical software package for agricultural research workers In: Hooda DS Hasija RC, editors. *Recent advances in information theory; statistics & computer applications*. Department of Mathematics Statistics. CCS HAU; Hisar: (1998). 139–43.

[44] Bajaj M. Nutrients and antinutrients in rice bean (*Vigna umbellata*) varieties as effected by soaking and pressure cooking. Asian J Dairy Food Res. (2014) 33:71–4. doi: 10.5958/j.0976-0563.33.1.015

[45] Siddhuraju P Osoniyi O Makkar HPS Becker K. Effect of soaking and ionising radiation on various antinutritional factors of seeds from different species of an unconventional legume, Sesbania and a common legume, green gram (*Vigna radiata*). Food Chem. (2002) 79:273–81. doi: 10.1016/S0308-8146(02)00140-1

[46] Katoch R. Nutritional and anti-nutritional constituents in different seed components of rice bean (*Vigna umbellata*). Indian J Agric Biochem. (2011) 24:65–7.

[47] Katoch R. Nutritional evaluation, protein digestibility and profiling of different Vigna species. Indian J Agric Biochem. (2013) 26:32–5.

[48] Shweta KR. Comparison of nutritional composition of Vigna spp. prevalent in the mid-hill region of Himachal Pradesh. Indian J Agric. Biochemist. (2014) 27:202–7.

[49] Dhingra D Michael M Rajput H. Dietary fibre in foods: a review. J Food Sci Technol. (2012) 49:255–66. doi: 10.1007/s13197-011-0365-5, PMID: 23729846 PMC3614039

[50] Fernandez ML Berry J. Nutritional evaluation of chick pea and germinated chickpea flours. Plant Foods Hum Nutr. (1988) 38:127–34. doi: 10.1007/BF01091717, PMID: 3200798

[51] Kataki P Deka SC Kotoki D Saikia S. Effect of traditional methods of processing on the nutrient contents and some antinutritional factors in newly developed cultivars of green gram (*Vigna radiata*.(L.)Wilezeh) and black gram (*Vigna mungo* (L.) Hepper) of Assam, India. Int Food Res J. (2010) 17:377–84.

[52] Khattak AB Klopfenstein C. Effects of gamma irradiation on the nutritional quality of grain and legumes I. stability of niacin, thiamine and riboflavin. Cereal Chem. (1989) 66:169–70.

[53] Rajyalakshmi P Geervani P. Nutritive value of the foods cultivated and consumed by the tribal South India. Plant Foods Hum Nutr. (1994) 46:53–61. doi: 10.1007/BF01088461, PMID: 7971787

[54] Arinathan V Mohan V Maruthupandian A Athiperumalsami T. Chemical evalution of raw seeds of certain tribal pulses in Tamil Nadu, India. Trop Subtrop Agroecosys. (2009) 10:287–94.

[55] Dahiya PK Linnemann AR Van Boekel MAJS Khetarpaul N Grewal RB Nout MJR. Mung bean: technological and nutritional potential. Crit Rev Food Sci Nutr. (2015) 55:670–88. doi: 10.1080/10408398.2012.671202 24915360

[56] Tswanya MN Kyuka C. Response of ten winged bean accessions on nutritional quality and nutrient uptake in the Guinea Savannah zone of north central, Nigeria. Asian J Food Res Nutr. (2022) 9:291–7. doi: 10.14738/assrj.96.12417

[57] Mieners C Derise N Lau HC Crews MG Ritchey SJ Murphy EW. The content of nine mineral elements raw and cooked mature dry legumes. J Agric Food Chem. (1976) 24:1126–30. doi: 10.1021/jf60208a036, PMID: 1002894

[58] Herrera-Hernández IM Armendáriz-Fernández KV Muñoz-Márquez E Sida-Arreola JP Sánchez E. Characterization of bioactive compounds, mineral content and antioxidant capacity in bean varieties grown in semi-arid conditions in Zacatecas, Mexico. Foods. (2018) 7:199. doi: 10.3390/foods7120199, PMID: 30563077 PMC6306736

[59] Makeri MU Mohamed SA Karim R Ramakrishnan Y Muhammad K. Fractionation, physicochemical, and structural characterization of winged bean seed protein fractions with reference to soybean. Int J Food Prop. (2017) 20:2220–36. doi: 10.1080/10942912.2017.1369101/SUPPL_FILE/LJFP_A_1369101_SM8294.DOCX

[60] Idowu AO Alashi AM Nwachukwu ID Fagbemi TN Aluko RE. Functional properties of sesame (*Sesamum indicum* Linn) seed protein fractions. Food Prod Process Nutr. (2021) 3:1–16. doi: 10.1186/S43014-020-00047-5

[61] Hahn M Darvill A Albersheim P. Host-pathogen interactions: XIX. The endogenous elicitor, a fragment of a plant cell wall polysaccharide that elicits phytoalexin accumulation in soybeans. Plant Physiol. (1981) 68:1161–9. doi: 10.1104/pp.68.5.1161, PMID: 16662068 PMC426062

[62] Jood S Chauhan B Kapoor A. Protein digestibility (in vitro) of chickpea and blackgram seeds as affected by domestic processing and cooking. Plant Foods Hum Nutr. (1989) 39:149–54. doi: 10.1007/BF01091894, PMID: 2762243

[63] Salunkhe DK. Legumes in human nutrition: current status and future research needs. Curr Sci. (1982) 57:387–94.

[64] Shet MS Murugiswamy B Madaiah M. Lipid profile and fatty acid composition of moth bean (Vigna aconitefolia) seeds. Fette, Seifen, Anstrichm. (1986) 88:264–6. doi: 10.1002/LIPI.19860880706

[65] Pattanayak A Roy S Sood S Iangrai B Banerjee A Gupta S. Rice bean: a lesser known pulse with well-recognized potential. Planta. (2019) 250:873–90. doi: 10.1007/s00425-019-03196-1, PMID: 31134340

[66] Caprioli G Giusti F Ballini R Sagratini G Vila-donat P Vittori S. Lipid nutritional value of legumes: evaluation of different extraction methods and determination of fatty acid composition. Food Chem. (2016) 192:965–71. doi: 10.1016/j.foodchem.2015.07.102, PMID: 26304436

[67] East J Nakayama T Parkman S. Changes in stachyose, raffinose, sucrose and monosaccharides during germination of soybeans. Crop Sci. (1972) 12:7–9. doi: 10.2135/cropsci1972.0011183X001200010003x

[68] Rupérez P. Oligosaccharides in raw and processed legumes. Eur Food Res Technol. (1998) 206:130–3. doi: 10.1007/s002170050228

[69] Han IH Baik BK. Oligosaccharide content and composition of legumes and their reduction by soaking, cooking, ultrasound, and high hydrostatic pressure. Cereal Chem. (2006) 83:428–33. doi: 10.1094/CC-83-0428

[70] Oatway L Vasanthan T Helm J. Phytic acid: a review. Food Rev Int. (2001) 17:419–31. doi: 10.1081/FRI-100108531

[71] Kataria A Chauhan B Punia D. Antinutrients and protein digestibility (in vitro) of Mungo bean as affected by domestic processing and cooking. Food Chem. (1989) 32:9–17. doi: 10.1016/0308-8146(89)90003-4

[72] Vijayakumari K Siddhuraju P Janardhanan K. Effects of various water or hydrothermal treatments on certain antinutritional compounds in the seeds of the tribal pulse, *Dolichos lablab* var. vulgaris L. Plant Foods Hum Nutr. (1995) 48:17–29. doi: 10.1007/BF01089196 8719735

[73] Janardhanan K Gurumoorthi P Pugalenthi M. Nutritional potential of five accessions of a south Indian pulse, *Mucuna pruriens* var utilis I. the effects of processing methods on the content of L-DOPA, phytic acid and oligosaccharides. Trop Subtrop Agroecosys. (2003) 1:141–52.

[74] Kamatchi Kala B Kalidass C Mohan V. Nutritional and antinutritional potential of five accessions of a south Indian tribal pulse Mucuna atropurpurea DC. Trop Subtrop Agroecosys. (2010) 12:339–52.

[75] Suneja Y Kaur S Gupta AK Kaur N. Levels of nutritional constituents and antinutritional factors in black gram (*Vigna mungo* L. Hepper). Food Res Int. (2011) 44:621–8. doi: 10.1016/j.foodres.2010.12.020

[76] Udayasekhara Rao P Deosthale YG. Tannin content of pulses: varietal differences and effect of germination and cooking. J Sci Food Agric. (1982) 33:1013–6. doi: 10.1002/jsfa.2740331012

[77] Khabiruddin MD Gupta SN Tyagi C. Nutritional composition of some improved genotypes of ricebean (*Vigna umbellata*). Forage Res. (2002) 28:104–5.

[78] Xu S Qin L Mazhar M Zhu Y. Functional components profile and glycemic index of kidney beans. Front Nutr. (2022) 9:1–10. doi: 10.3389/fnut.2022.1044427, PMID: 36407530 PMC9667044

[79] Yao Y Cheng XZ Wang LX Wang SH Ren G. Major phenolic compounds, antioxidant capacity and antidiabetic potential of rice bean (*Vigna umbellata* L.) in China. Int J Mol Sci. (2012) 13:2707–16. doi: 10.3390/ijms13032707, PMID: 22489119 PMC3317682

[80] Amin HAS Hanna AG Mohamed SS. Comparative studies of acidic and enzymatic hydrolysis for production of soyasapogenols from soybean saponin. Biocatal Biotransformation. (2011) 29:311–9. doi: 10.3109/10242422.2011.632479

[81] Difo VH Onyike E Ameh DA Njoku GC Ndidi US. Changes in nutrient and antinutrient composition of Vigna racemosa flour in open and controlled fermentation. J Food Sci Technol. (2015) 52:6043–8. doi: 10.1007/s13197-014-1637-7, PMID: 26345026 PMC4554638

[82] Sekhon J Grewal S Singh I Kaur J. Evaluation of nutritional quality and antioxidant potential of pigeonpea genotypes. J Food Sci Technol. (2017) 54:3598–611. doi: 10.1007/s13197-017-2818-y, PMID: 29051655 PMC5629169

[83] Nisar J Shah SMA Akram M Ayaz S Rashid A. Phytochemical screening, antioxidant, and inhibition activity of Picrorhiza kurroa against α-amylase and α-glucosidase. Dose-Response. (2022) 20:1–12. doi: 10.1177/15593258221095960, PMID: 35558871 PMC9087273

[84] Di SE Oliviero T Udenigwe CC. Functional significance and structure–activity relationship of food-derived α-glucosidase inhibitors. Curr Opin Food Sci. (2018) 20:7–12. doi: 10.1016/j.cofs.2018.02.008

[85] Spencer JW Ethan DGB. α-Glucosidase inhibitors as host-directed antiviral agents with potential for the treatment of COVID-19. Biochem Soc Trans. (2020) 48:1287–95. doi: 10.1042/BST20200505 32510142

[86] Savage G Deo S. The nutritional value of peas (*Pisum sativum*): a literature review. Nutr Abstr Rev Ser A. (1989) 59:66–88.

[87] Khalyfa A Kermasha S Alli I. Partial purification and characterization of lipoxygenase of canola seeds (Brassica napur var. wester). J Agric Food Chem. (1990) 38:2003–8. doi: 10.1021/jf00101a003

[88] Katoch R. Incriminating factors in rice bean (*Vigna umbellata*) and processing techniques for their minimization. Indian J Agric Biochem. (2014) 27:138–44.

[89] Diaz S Polania J Ariza-Suarez D Cajiao C Grajales M Raatz B. Genetic correlation between Fe and Zn biofortification and yield components in a common bean (*Phaseolus vulgaris* L.). Front Plant Sci. (2022) 12:1–13. doi: 10.3389/fpls.2021.739033, PMID: 35046970 PMC8761845

[90] Welch RM Graham RD. Breeding for micronutrients in staple food crops from a human nutrition perspective. J Exp Bot. (2004) 55:353–64. doi: 10.1093/JXB/ERH064 14739261

